# Increased liver injury in patients with chronic hepatitis and IgG directed against hepatitis E virus

**DOI:** 10.17179/excli2019-1827

**Published:** 2019-10-25

**Authors:** Daouda Sévédé, Moussa Doumbia, Viviane Kouakou, Vicky Djehiffe, Pascal Pineau, Mireille Dosso

**Affiliations:** 1 Département de Bactériologie Virologie, Institut Pasteur de Côte d'Ivoire, Abidjan, Côte d'Ivoire; 2 Laboratoire de Sérologies bactériennes et virales, Institut Pasteur, Abidjan, Côte d’Ivoire; 3 Unité "Organisation nucléaire et Oncogenèse", INSERM U993, Institut Pasteur, Paris, France

**Keywords:** hepatitis E virus, seropositivity, aggravation of liver damage, Côte d'Ivoire

## Abstract

Type-E hepatitis is responsible for more than three million symptomatic cases and more than 40,000 deaths worldwide. The situation of this hepatitis is overall poorly known in sub-Saharan Africa. Notably, the baseline circulation of HEV outside sporadic outbreaks has been barely characterized in this large region. More specifically, the impact of superinfection by this virus on the health status of the large reservoir of patients chronically infected with other hepatitis viruses remains to be evaluated. We searched for anti-HEV immunoglobulins in a series of 200 pregnant women and 92 patients with persistent liver infections with hepatitis B or C viruses and subsequently tried to assess serological co-variations with demographical and clinical features. We observed that only 1.5 % of expectant mothers were seropositive of anti-HEV IgG while it was the case for 18.4 % of patients with chronic liver diseases (P=4.5E-07). The presence of anti-HEV was not linked to any of the collected demographical features (age, sex, education, pork meat consumption, water supply, …). By contrast, the presence of anti-HEV was significantly associated with increased levels (1.6-1.8-fold, P<0.0001) of blood aminotransferases (AST, ALT) in patients with persistent hepatitis B or C. Our work indicates that, in Ivory Coast, the presence of IgG directed against HEV might contribute to a deterioration of liver health in patients with already installed persistent liver infections. The mechanisms explaining such phenomenon at distance of acute phase of infection are still unknown but might be linked either to a residual persistence of HEV in a context of general immune exhaustion or to an inappropriate auto-immune reaction as already observed in the aftermath of other viral infection types.

## Introduction

Infection with the hepatitis E virus (HEV) has so far been considered as permanently resolving in the immense majority of cases. Acute hepatitis E takes a cholestatic presentation in 10 % of patients and a high mortality rate (20 %) is, for unexplained reasons, restricted to pregnant women. The severity of infection with HEV is correlated with the age of the patient although effectors of unfavorable evolution are still poorly understood. Recent studies, however, have shown that a progression towards chronicity defined by the persistence of viremia for more than six months is possible notably in the case of immune suppression (Péron et al., 2006[19]; Tamura et al., 2007[23]). The level of immune suppression seems to play a major role in the installation of HEV persistence but no study has shown a predominant defect either in the innate or in the adaptive response. In some chronically HEV-infected patients, rapid progression to liver cirrhosis in less than three years has been described (Gérolami et al., 2008[11]; Kamar et al., 2008[15]). 

Concerning the serological response to HEV infection, antibodies directed against the virus (IgG and IgM types) are detectable from the beginning of the symptomatology with a maximum rate of positivity after one month and a decrease after 2 to 6 months for IgM. Type G immunoglobulins are known to persist from 18 months to more than 10 years. Most studies conducted in adults have shown that seroprevalence of anti-HEV varies from 10 to 50 % depending on the geographical origin of the populations. The purpose of this study, which has never been done in my country, was to determine for the first time the prevalence of type E viral hepatitis; suspected to increase morbidity and mortality in persistently infected Ivorian patients with hepatitis B virus (HBV), hepatitis C virus (HCV) or human immunodeficiency virus (HIV) and in subjects at risk such as pregnant women.

## Patients and Methods

This work is a cross-sectional study conducted on 292 patients at the Institut Pasteur of Côte d'Ivoire from January 2016 to December 2018. The study was approved by the National Committee of Ethics and Research (Ref#047/MSHP/CNER.Kp) and is conform to the declaration of Helsinki. Informed consent was obtained from all subjects (or their tutors if they were children) who participated in the study. A structured questionnaire was then administered via a case report form. A blood sample (approximately 5 ml) was taken at the elbow fold after local disinfection. The samples were centrifuged at 5 min at 3000 rpm to separate serum from blood cells. The serum was stored at -20° C until biological analyses. Expectant mothers were randomly recruited at the sampling center associated to the medical laboratories of the Institut Pasteur de Côte d'Ivoire. The subjects with liver diseases corresponded to patients received in the same facility in the frame of initial diagnosis of persistent infection.

Samples were analyzed for HBsAg and anti HCV assays using ARCHITECT plus i1000 (Abbott. Chicago, IL, USA) and COBAS 6000 (Roche, Abidjan, Côte d'Ivoire) automated machines. HIV was searched according to the national algorithm using Determine HIV1/HIV2 (Abbott, Chicago, IL, USA) and Bio-Line HIV1-2 (Standard Diagnostics, Yongin, South Korea). Anti-HVE IgG type and the anti-HVE type IgM Ab were assessed by ELISA using the HEV kit IgG-IgM (Wantai Bio-Pharm, Beijing, PR of China). 

Data were stored and subsequently analyzed by the software Epi-info 7.1.3.3. Fisher Exact test was used to compare the categorical variables while Student t test, Mann-Whitney U test or ANOVA test were used for continuous variables as appropriate. The minimal significance threshold was set at P < 0.05.

## Results

A total of 292 patients were enrolled in the study including 200 pregnant women attending a prenatal check-up and 92 patients followed for chronic liver infection in Cocody University hospital of Abidjan. The demographical and clinical characteristics of the patients are included in Table 1(Tab. 1). In brief, pregnant women were, as expected, significantly younger than patients with persistent viral infection (29.5 ± 6.5 years vs 43.1 ± 16.1 years, P<1.0E-04). Ethno-anthropological backgrounds of both groups were not different (P>0.05, ns). Pregnant women tend to display a lower education level (P=0.004) with one**-**third of them who did not benefit from any schooling. Regarding marital status, a more important proportion of patients with liver disease were single (32.6 % vs 18.0 %, P=9.6E-09). Enrolled subjects were mostly Abidjan dwellers (87-94 %) in both subsets but indices vs 1.8 ± 0.7 P=3.7E-04) with chronically-infected patients living in more crowded housings. Proportions of pork consumers and sources of potable water supplies were not different between the groups.

Regarding persistent infections, expectant mothers displayed rather low rates of HBV (4.0 %), HCV (0.0 %) or HIV (0.5 %) infections whereas serological scars for mono-infections with HBV or HCV or dual HBV/HCV infections were found in similar proportions (32.6-34.7 %) in patients with viral persistence (Table 2(Tab. 2)). HIV was also present in a small subset of patients with hepatitis (6.5 %). Blood aminotransferases concentrations were of course significantly different (P<1.0E-30) between groups. Concerning seroreactivity against HEV, type G immunoglobulins were the only detectable subtypes. Their prevalence was significantly higher in chronically infected patients than in pregnant women (18.4 % vs 1.5 %, OR=14.7, 95 % CI=4.1-80.6, P=4.5E-07). 

When considering only the patients with a previously known liver disease, the age of anti-HEV(+) patients were somewhat higher, albeit non significantly than the age of non-seropositive ones (47.2 ± 17.5 years vs 42.1 ± 15.5 years, ns). No demographical or lifestyle features, including pork consumption or potable water supply, were differing between anti-HEV seropositive and seronegative patients. The presence of anti-HEV was not associated with any other virus (HBV, HCV, HIV) in particular.

**Figure 1:**
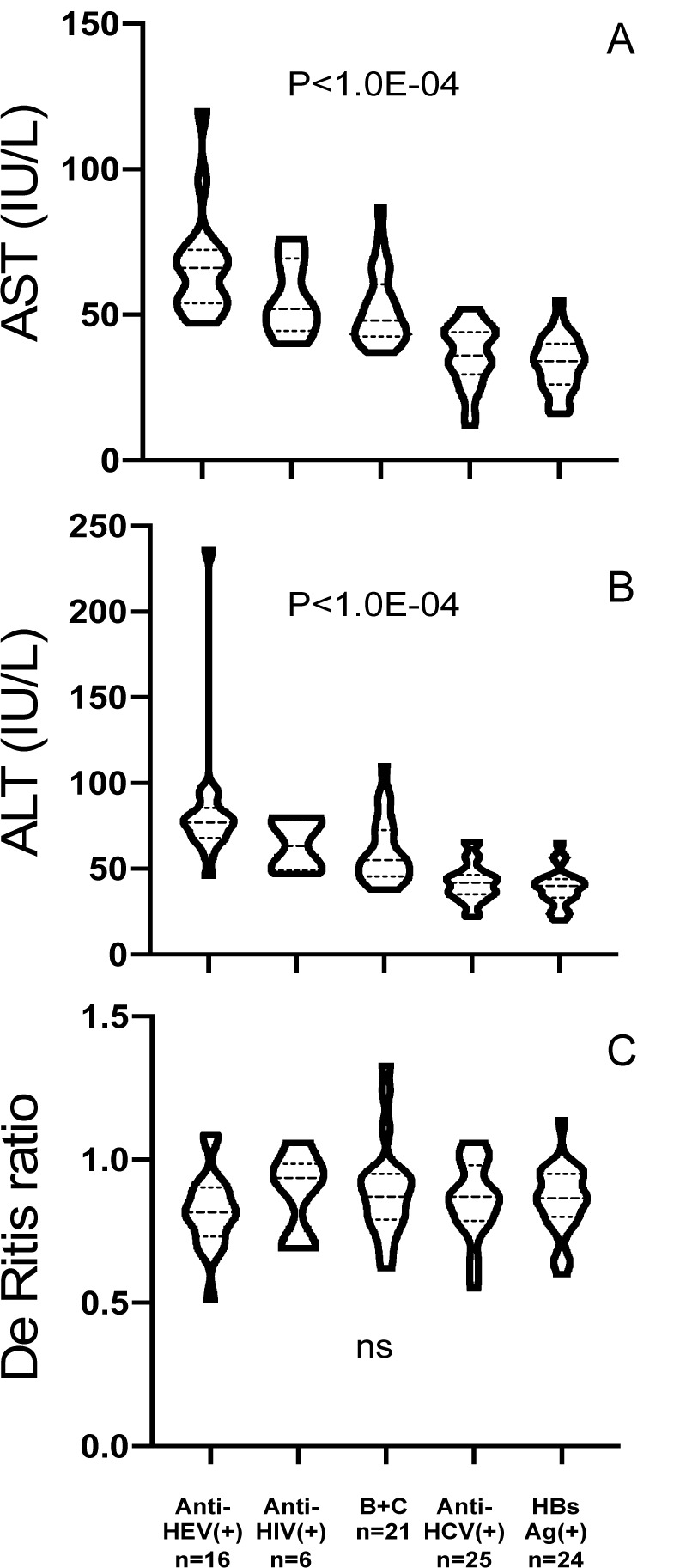
Levels of aspartate aminotransferase (AST), alanine aminotransferase (ALT), and De Ritis ratio (AST/ALT) as measured for the different group of patients with a persistent liver infection.

Remarkably, liver enzymes concentrations were significantly increased in anti-HEV(+) when compared to anti-HEV(-) in patients with persistent infections (Figures 1A and 1B(Fig. 1)). This observation was slightly more pronounced in the case of ALT (1.8-fold) than for AST (1.6-fold). By contrast, de Ritis ratios (AST/ALT) were not significantly different (0.81 ± 0.13 vs 0.87 ± 0.13, P=0.13, ns) between anti-HEV(+) and corresponding seronegative patients (Figure 1C(Fig. 1)). Remarkably, aminotransferases values were still significantly higher in anti-HEV(+) than in case of dual HBV-HCV infection supposedly associated with a more severe liver injury (P=0.0076 and P=0.0191 for AST and ALT respectively, Figures 1A and 1B(Fig. 1)). 

This observation suggests a real long-term deleterious effect of past Type-E hepatitis on liver tissue of patients already chronically infected with viruses commonly responsible for persistent infection.

## Discussion

In the absence of immune suppression, infection with HEV is considered to be controlled without any sequel in most cases, to the notable exception of pregnant women representing an intriguing group of vulnerable patients. Recent data produced both in Eastern Asia or sub-Saharan Africa, however, indicate that HEV infection might represent a risk factor of chronic liver disease progression leading ultimately to hepatocellular carcinoma (HCC) (Amanya et al., 2017[2]; Amougou Atsama et al., 2017[3]; Bai et al., 2018[6]; Diwe et al., 2013[9]).

In the present series, carriage of anti-HEV IgG was not significantly associated with any of the commonly described risk factors such as age, or pork meat consumption (Cook et al., 2017[8]; Hartl et al., 2016[13]). 

Seroreactivity against HEV was rather associated with a deterioration of biological chemistry features such as aminotransferases. 

The presence of anti-HEV IgG without a trace of corresponding IgM in the present series indicates that all infections were rather distant from the time of sampling and that we were thus not in the presence of acute type E hepatitis. Nevertheless, in a series of patients already affected by a persistent viral infection of the liver either with HBV or HCV, those patients carrying anti-HEV IgG were displaying higher mean plasma values for both liver aminotransferases suggesting a more severe injury process. This observation was not merely resulting from mean values differences but was also validated using the ranking of patients. Indeed, twelve and thirteen anti-HEV patients (70.5 %, n=12/17, and 76.4 %, n=13/17) were positioned in the higher quartiles for AST and ALT respectively.

The present study was not conducted in the context of an acute superinfection with HEV of patients with an already advanced liver disease, circumstances that understandably increase the risk of brutal decompensation and death (Amougou Atsama et al., 2017[3]; Hamid et al., 2002[12]; Radha Krishna et al., 2009[21]). Our work was instead conducted in the frame of a generally mild chronic liver disease (initial diagnosis) and a context of already distant infection with HEV (presence of IgG, absence of IgM). Despite, this apparently more favorable context, we observed an ongoing more severe liver injury process in anti-HEV carriers. A higher prevalence of anti-HEV IgG in patients with chronic liver disease of another etiology had been already recognized in different parts of the world (Atiq et al., 2009[4]; Cheng et al., 2013[7]; Kondili et al., 2006[16]), however they have been rarely associated with abnormal liver function tests and considered, as such, as indicative of liver disease progression (Amougou Atsama al., 2017[3]; Hoan et al. 2015[14]).

The causes of this phenomenon are difficult to explain. We hypothesize that increased liver injury could be generated by two different processes. First, in a context of immune exhaustion due to a long-term liver infection either with HBV or HCV, HEV might persist in rare hepatocytes and stimulate an additional layer of inefficient but deleterious immune response. The liver is considered as an immune tolerant tissue, but, nevertheless, low-level infections such as occult B infection have been associated with serious liver diseases such as cirrhosis or even HCC (Pollicino et al., 2004[20]). Other viruses, including RNA viruses (HCV, measles virus) are known to generate low levels of persistent infections with serious consequences (Alazawi et al., 2010[1]; Noubiap et al., 2015[18]; Rima and Duprex, 2005[22]). Such type of persistence has not been observed so far but it is doubtful that this hypothesis has been explored with sufficient conviction. The second mechanism could be the generation of an auto-immune response directed against some liver-specific proteins subsequently to acute Type-E hepatitis on chronic type B or C hepatitis. It is well known that many different viral infections are the cause of auto-immune reactions with pathological consequences (Bach, 2005[5]; Newton et al., 2015[17]).

We are aware that our study suffers from several shortcomings. The main one concerns the number of participants that locates in the lower range for studies of this type. Other limitations are concerning serological or viral features such as nucleic acid detection for HBV or HCV that were unavailable in the present work. Finally, a control group including patients with anti-HEV only might have shed a useful light on the synergism between HEV and other hepatitis viruses. We consider, however, that accumulated evidence reasonably supports the role of HEV in the deterioration of the liver stratus of Ivorian patients affected from Type-B or -C persistent infection. As such, the present work should be primarily considered as a pilot exploration that deserves followers.

## Conclusion

In conclusion, we observed that the past Type-E hepatitis materialized by the presence of anti-HEV IgG in African patients with chronic hepatitis B or C appears to be responsible for a substantial increase of liver injury (Feldt et al., 2013[10]). The long-term consequences of this situation in terms of severe complications such as liver cirrhosis or HCC are unknown. Further surveys are warranted to confirm and deepen the extent of our knowledge about the role HEV infection in the context of sub-Saharan African countries with a large segment of the population chronically infected with hepatitis B or C viruses.

## Ethical approval and consent to participate

The study was approved by the National Committee of Ethics and Research (Ref#047/MSHP/CNER.Kp) and is conform to the declaration of Helsinki. Informed consent was obtained from all subjects (or their tutors if they were children) who participated in the study. 

## Competing interests

The authors declare no conflict of interest.

## Funding

Personal financing

## Authors' contributions

DS conceived the research project, recruited the patients, collected the data, performed the experiments, analyzed data and produced the first draft. MD performed experiments and edited the manuscript. VK, and VD performed serological tests. PP analyzed the data and edited the manuscript. MD edited the manuscript.

## Acknowledgements

We would like to thank our colleagues in the Institut Pasteur Côte d’Ivoire and the Institut Pasteur in Paris for their constant support.

## Figures and Tables

**Table 1:**
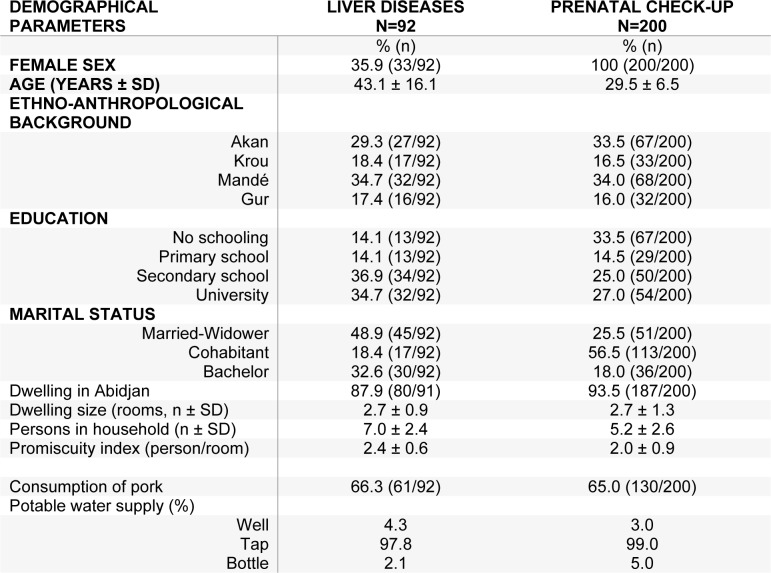
Frequency of the demographic and clinical characteristics of the patients included in the study

**Table 2:**
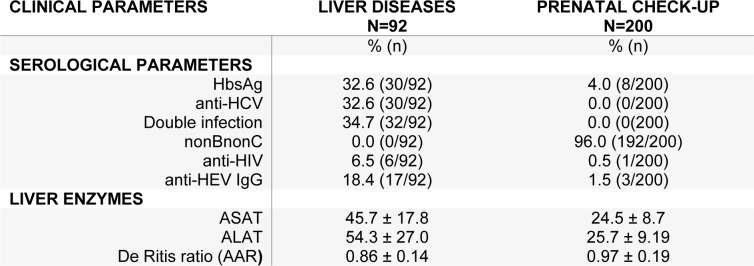
Frequency of the demographic and clinical characteristics of the patients included in the study
